# Influence of prosthesis design and implantation technique on implant stresses after cementless revision THR

**DOI:** 10.1186/1749-799X-6-20

**Published:** 2011-05-13

**Authors:** Markus O Heller, Manav Mehta, William R Taylor, Dong-Yeong Kim, Andrew Speirs, Georg N Duda, Carsten Perka

**Affiliations:** 1 Julius Wolff Institute and Center for Musculoskeletal Surgery Charité - Universitätsmedizin Berlin, Germany

**Keywords:** revision hip arthroplasty, implant stresses, implant design, surgical technique, physiological loading, computational modelling

## Abstract

**Background:**

Femoral offset influences the forces at the hip and the implant stresses after revision THR. For extended bone defects, these forces may cause considerable bending moments within the implant, possibly leading to implant failure. This study investigates the influences of femoral anteversion and offset on stresses in the Wagner SL revision stem implant under varying extents of bone defect conditions.

**Methods:**

Wagner SL revision stems with standard (34 mm) and increased offset (44 mm) were virtually implanted in a model femur with bone defects of variable extent (Paprosky I to IIIb). Variations in surgical technique were simulated by implanting the stems each at 4° or 14° of anteversion. Muscle and joint contact forces were applied to the reconstruction and implant stresses were determined using finite element analyses.

****Results**:**

Whilst increasing the implant's offset by 10 mm led to increased implant stresses (16.7% in peak tensile stresses), altering anteversion played a lesser role (5%). Generally, larger stresses were observed with reduced bone support: implant stresses increased by as much as 59% for a type IIIb defect. With increased offset, the maximum tensile stress was 225 MPa.

****Conclusion**:**

Although increased stresses were observed within the stem with larger offset and increased anteversion, these findings indicate that restoration of offset, key to restoring joint function, is unlikely to result in excessive implant stresses under routine activities if appropriate fixation can be achieved.

## Background

The total number of revision joint replacement surgeries is expected to increase as a result of an aging population and because of wider surgical indications for primary implantation [1]. There are, however, only limited options for revision of the femoral component in the presence of an extensively compromised bone stock, and there is no consensus as to the best option for fixation of the femoral component under such difficult conditions [2,3]. Successful femoral reconstruction requires a femoral component that will be axially and rotationally stable and restores femoral offset and femoral anteversion.

The Wagner SL revision stem is a cementless component that allows the mechanically incompetent proximal femur to be bypassed. The tapered design allows for a distal fixation and longitudinal flutes provide rotational stability [4]. The initial design of the stem has been shown to produce good short to mid-term clinical results [5-7] and clinical follow-ups have demonstrated the success of the implant in bridging extended femoral bone defects [8,9]. However, there have been a number of cases where failures have been reported due to dislocations [7,10], and it has been speculated whether the dislocation rate for this specific stem could be linked to the rather small femoral offset of the original prosthesis design.

It is known that reconstruction of the femoral offset is crucial for obtaining proper joint function [11] and stability [12] in total joint replacements [13,14], especially in revision patients with potentially reduced soft tissue tension due to insufficient gluteal musculature [15]. It therefore seems desirable to implant a prosthesis with a sufficient offset to reduce the risk of early dislocations in patients with anatomically larger offsets or laxity of the abductor muscles, but such geometrical modifications are known to affect the loads acting on the reconstruction [16]. Although an increased offset results in reduced hip contact forces due to an increase in the lever arms of the abductors, it could also result in larger implant stresses due to increased bending moments, specifically in extended defects, where only a rather distal diaphyseal implant fixation can be achieved [17].

In addition to the offset, femoral anteversion is a key factor that has been shown to affect both the dislocation rate [18] and the forces acting across the hip [19] but might be difficult to control precisely. Due to the rather complex interactions between joint geometry as defined by e.g. the combination of femoral offset and anteversion, and the resulting musculoskeletal loading conditions, it is not readily apparent whether a prosthesis design with an increased offset would be linked to only decreased muscle and joint contact forces and potentially improved joint function or whether increased stem stresses and eventual implant failure become possible consequences.

Validated musculoskeletal analyses can determine the in vivo loads acting in the lower limb [20], as well as the influence of alterations of hip joint geometry on the resulting forces across the joint [19]. Furthermore, finite element analyses that apply physiological-like loading conditions are capable of assessing the straining in the healthy femur as well as the load sharing conditions after reconstruction [21,22]. By applying a combination of these techniques, it seems possible to investigate how specific combinations of design and surgeon related factors might interact and whether certain combinations are likely to result in mechanical conditions that might challenge the survival of the reconstructed joint [22,23].

The goal of the current study was therefore to understand the load transfer from the implant to the bone after revision of the femoral component with distal bone anchorage and in the presence of a compromised bone stock, as well as the influence of increased offset on the implant stresses under these conditions. Specifically, we tested the hypothesis that an increased offset, an increased anteversion, or their combination, would result in increased implant stresses, particularly in large bone defects.

## Materials and methods

### Solid model

**Figure 1:**
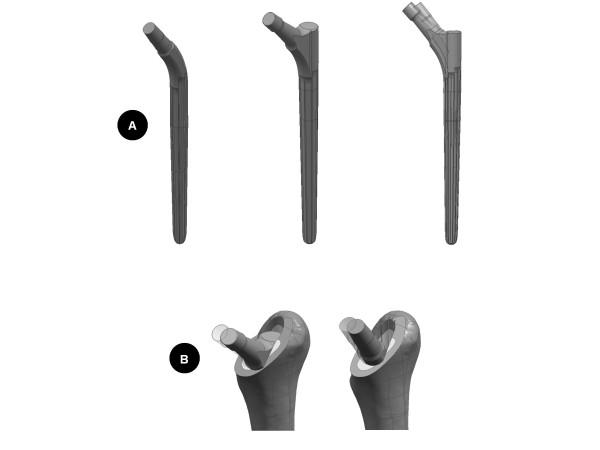
**Prosthesis designs and their implantations**. A (top): Two different designs of the Wagner revision stem. *Left: *34 mm offset prosthesis (standard prosthesis). C*entre: *44 mm offset prosthesis (increased offset prosthesis). *Right*: Superposition of the two stem designs, with the standard prosthesis shown as translucent. B (bottom): Variation of surgical implantation. *Left: *44 mm offset stem implanted at 4° (transparent) and 14° of femoral anteversion, *Right: *34 mm offset stem implanted at 4° (transparent) and 14° of femoral anteversion.

Solid models of the Wagner SL cementless femoral revision stem were obtained from the manufacturer (Zimmer GmbH, Winterthur, Switzerland, Figure 1). Two prosthetic designs were investigated: the standard prosthesis (34 mm offset) and an increased offset design (44 mm offset). To study the influence of surgical technique, both stem designs were implanted virtually with 4 or 14 degrees of anteversion (Figure 1) into a solid model of the Standardized Femur following the manufacturers recommended technique. Thereby, the influence of both design and surgical technique on implant stresses was characterized and compared between four models.

### Musculoskeletal analysis

Based on a previously validated musculoskeletal model of the lower limb [20], muscle and joint contact forces were derived and subsequently applied to the finite element models [22]. In brief, the muscle attachment sites and joint coordinates were obtained from the visible human and then scaled to fit the anatomy of the Standardized femur (CT-data, Visible Human, NLM, USA). The muscle paths were modelled as straight lines from origin to insertion sites, wrapping around the bone to represent the more realistic curved paths of the muscles. The physiological cross-sectional area of each muscle was determined from the literature and scaled to fit an assumed body weight of 820N. Inverse dynamics calculations based on measured forces from gait cycles of a patient were used to determine intersegmental resultant forces for the Standardized Femur geometry. Static optimisation was performed to minimize sum of the square of the muscle stresses [24]. A balanced set of muscle and joint contact forces was therefore determined and applied for each finite element model configuration (Table 1, Table 2).

**Table 1:** Three-dimensional hip contact force components [N] during normal walking, as applied to the finite-element-models for each of the four different implantation configurations.

Implantation Configuration	Hip Contact Force Component		
	x	y	z
**A**: 34 mm offset, 4° anteversion	-611	-73	-2539
**B**: 44 mm offset, 4° anteversion	-659	-100	-2449
**C**: 34 mm offset, 14° anteversion	-639	-4	-2679
**D**: 44 mm offset, 14° anteversion	-694	-24	-2592

Positive force components act medial (+x), anterior (+y), superior (+z). A, B, C, D represent the four implant configurations.

**Table 2:** Muscle forces [N] applied in the finite-element-analyses for each of the four different implantation configurations (A to D, compare Table 1).

		Muscle Force		
Muscle	A	B	C	D
*Gluteus maximus part 1*	202.1	181.8	161.4	139.8
*Gluteus maximus part 2*	48.1	39.8	37.0	30.2
*Gluteus maximus part 3*	126.7	163.1	126.1	163.4
*Gluteus medius part 1*	251.6	241.7	221.9	213.1
*Gluteus medius part 2*	130.9	136.5	122.7	128.3
*Gluteus medius part 3*	267.6	294.1	261.2	285.6
*Gluteus minimus part 1*	19.0	18.9	17.2	17.2
*Gluteus minimus part 2*	32.8	34.7	31.1	32.9
*Gluteus minimus part 3*	65.8	75.9	65.6	74.9
*Pirirformis*	81.9	68.6	64.3	53.6
*Biceps femoris caput long*.	290.0	370.3	300.5	383.2
*Semitendinos us*	470.6	496.5	492.1	517.8
*Semimembranosus*	37.9	40.0	38.8	40.6
*Tensor fascia latae*	36.2	47.7	38.1	49.2
*Gastrocnemius lateralis*	7.8	13.2	8.6	13.9
*Biceps femoris caput brevis*	9.4	14.3	10.0	14.9
*Vastus intermedius*	442.9	456.3	448.1	460.9
*Vastus lateralis*	428.0	500.8	439.1	511.3
*Vastus medialis*	106.1	41.7	100.7	5.4

### Finite element models

Meshes for all components in the finite element models were generated using non-linear second order 10-node tetrahedral elements (Patran, MSC Software Corp, Santa Ana, CA, USA). Depending on the combination of prosthetic design and implantation, the developed models resulted in a total element count of up to 131,300.

**Figure 2:**
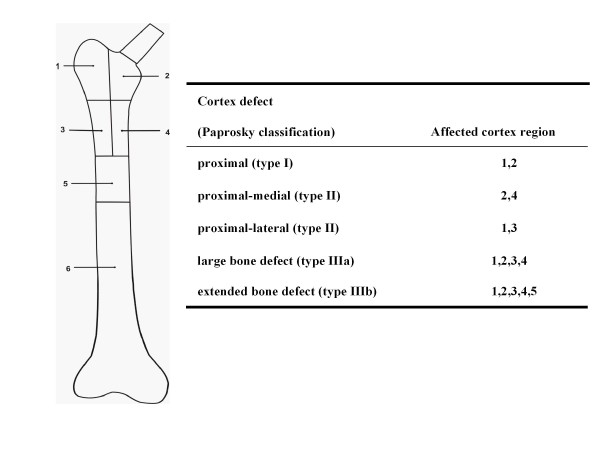
**Bone defect regions**. In order to assess the effect of different extents of femoral bone defect on implant loading, the femoral cortex was divided into a number of regions (medial, lateral, proximal, distal) according to the Paprosky classification (Paprosky et al., 1994). The material properties of the cortex were then reduced to simulate the effects of bone loss for each of the different defect situations.

The effect of bone defects was analysed by simulating the cortical thinning and bone loss conditions under which the Wagner SL stem might be used clinically. A total of five bone defects exhibiting different extents of bone loss were analysed (Figure 2): a proximal defect (type I, [25]), a proximal medial (type II), a proximal lateral (type II), a large bone defect (type IIIa), and an extended bone defect (type IIIb). The length of the largest defect (extended defect), starting from the tip of the greater trochanter measured 17.3 cms. In order to facilitate comparisons across the different defects, a single implant size (stem diameter) was used throughout. Here, the determination of the implant size was driven by the most extended defect that was anticipated to represent the worst case scenario in terms of implant stresses, and for which the stem size chosen was considered adequate.

**Figure 3:**
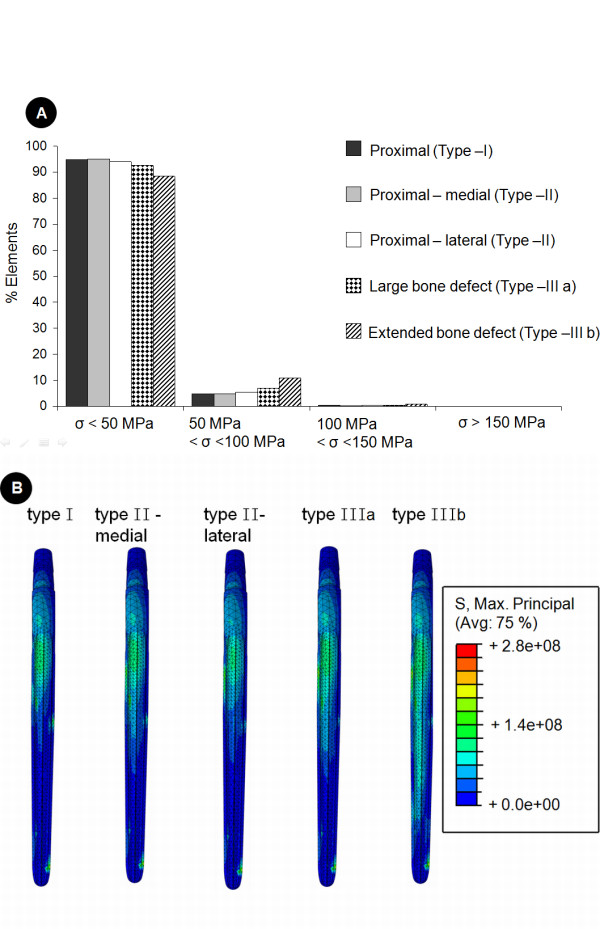
**Implant stresses within the standard design prosthesis as a function of the extent of the bone defect**. This figures shows the effect of the extent of bone defect on the tensile stresses within the standard prosthesis (34 mm femoral offset) implanted at 4° of femoral anteversion. In image A (top), a histogram of the implant stresses is shown. Here, for each bone defect simulation implant elements were grouped according to their maximum principle (i.e. tensile) stress (denoted by the symbol σ) and are presented as a percentage of the total number of elements in the implant. Image B (bottom) shows the stress distribution along the lateral aspect of the implant for bone defects of increasing extent.

In addition to removing the trabecular bone, the thinning of the cortex associated with this form of bone defect was simulated by reducing the material properties of specific regions of the cortex (Figure 3) to an elastic modulus of 5 GPa and a Poisson's ratio of 0.4. By using this reduced modulus but maintaining the intact bone's actual thickness, the resulting bending stiffness (second moment of area) in the coronal plane of the cortex was calculated to be equivalent to a 2 mm thin cortex with an elastic modulus of 17 GPa. The intact cortices of the bone (distal sections of the femur) were assigned an elastic modulus of 17 GPa (ν = 0.4) [26], while trabecular bone was modelled with an elastic modulus of 2 GPa (ν = 0.4). The titanium alloy Wagner SL revision stem was assigned an elastic modulus of 110 GPa, and a Poisson's ratio of 0.3.

Tied contact constraints were used over the distal anchorage, while the remaining contact surface areas of the prosthesis and bone interface were defined as frictionless sliding, using a modified formulation for the non-linear second order tetrahedrons. Nodes on the slave contact surface were initially adjusted to lie directly on the master surface without inducing any stresses or strains within either material.

To prevent rigid body motion, displacement constraints were applied to nodes at the centre of the knee, the location of the hip contact force and on the distal lateral surface of the lateral condyle [27]. Thus, three translational degrees of freedom were constrained at the knee; the hip was allowed to translate along the axis connecting the hip and knee; the node on the lateral condyle was constrained to prevent rotation of the model about the hip-knee axis.

Non-linear finite element analysis was performed using ABAQUS v6.5 (ABAQUS Inc., Providence, USA). Implant stresses were evaluated by querying the element centroids and grouped into element sets that corresponded to certain stress limits. The different bone defect models were then compared to determine the influence of offset and anteversion modifications on implant stresses.

## Results

For the 34 mm offset stem implanted at 4° of femoral anteversion, more than 88% of the implant model experienced tensile stresses that remained below 50MPa (Figure 3 A). The maximum tensile stress calculated within a single element of the implant for the case of a proximal (type I) defect was 141MPa.

### Influence of the extent of bone defect

In general, the implant stresses increased with progressing bone defect severity (Figure 3): while only 5% of the implant experienced stresses over 50MPa for a type I defect, over 12% of the implant was subjected to these stresses for the reconstruction of a type IIIb defect. For this extended type IIIb bone defect, peak stresses within the standard prosthesis (34 mm offset) increased by 59% when compared to the implant stresses for the proximal (type I) defect. The largest maximum principal (i.e. tensile) stresses were distributed along the lateral aspect of the shaft, and distal lateral side of the implant neck (Figure 3). When comparing proximal bone defects, bone loss on the medial side had a larger effect on the implant stresses than bone loss on the lateral side.

### Influence of prosthesis design

**Figure 4:**
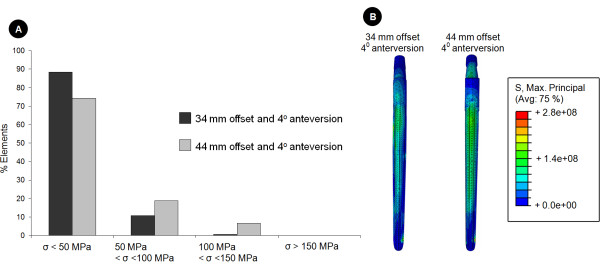
**Effect of design variation on the stress distribution in the implants**. For the situation of an extended bone defect (Paprosky type IIb) this figure demonstrates the effect of femoral offset on the maximum principle (i.e. tensile) stresses within the implant. The implant elements were grouped according to their stress (denoted by the symbol σ) level. This data is presented as a histogram in image A (top), where the data are reported as a percentage of the total number of elements in the implant. Below, image B compares the stress distributions along the lateral aspect of the implant for a type IIIb defect for the two different offsets. It can be seen that the implant with the increased offset experiences larger tensile stresses.

Increasing the neck length from 34 to 44 mm induced larger implant stresses (Figure 4). For situations with an extended bone defect (type IIIb), together with an increased offset (44 mm) prosthesis implanted at 4° femoral anteversion, more than 26% of the implant experienced tensile stresses of over 50MPa, while only 12% of the implant was subjected to such stresses for the standard offset. In this scenario, an upper stress limit of 225MPa was determined, which amounted to a 16.7% increase in peak tensile stresses in comparison to the same defect situation for the standard prosthesis. The stresses for the increased offset design appeared to be distributed further on the lateral aspect and distal neck of the implant.

### Influence of anteversion

**Figure 5:**
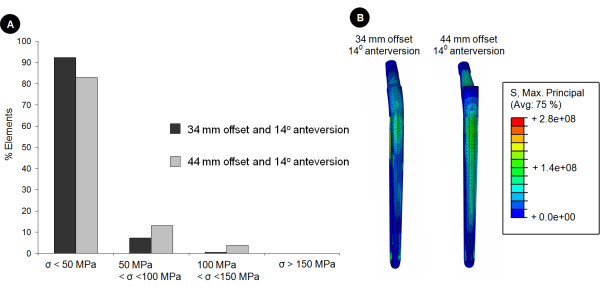
**Effect of surgical technique on the stress distribution within the implants**. For the situation of an extended bone defect (Paprosky type IIb) we further explored the effect of surgical technique (implantation) on the implant stresses by varying femoral anteversion and examining its effect on the maximum principle (i.e. tensile) implant stresses. Here, the implant stresses of the standard (34 mm) and increased offset (44 mm) prostheses implanted at 14° of femoral anteversion are compared. This data is again presented as a histogram in image A (top), with the results reported as a percentage of the total number of elements in the implant stressed within a certain stress level. Below, image B compares the stress distributions along the lateral aspect of the implant for a type IIIb defect for the two different offsets. It can be seen that also for 14° of femoral anteversion the implant with the increased offset experiences larger tensile stresses than the standard prosthesis.

Increasing the anteversion from 4° to 14° in the standard prosthesis (34 mm) resulted in an increase of approximately 5% in peak tensile stresses within the implant (Figure 5). However, implantation of the stem with an anteversion of 14°, together with a combined increase in offset (44 mm) caused almost a 15% increase in stresses within the implant when compared to the standard prosthesis (Figure 5).

## Discussion

By examining the effects of two different implant offsets and the variation of anteversion, this numerical analysis demonstrates that the stress levels developed within the Wagner SL revision stem are the highest in situations with severely compromised bone stock. A combination of increased offset and anteversion, resulted in the highest stresses, but even this combination should not induce critical stresses in the implant during normal activities of daily living, even for an extensive bone defect (Paprosky type IIIb), necessitating distal fixation.In all regions of the implant, the maximum determined stresses of 225MPa remained well below the implant material's fatigue limit of 450MPa [28], suggesting that the implant is capable of withstanding normal physiological loading without the risk of failure.

While in clinical practice the diameter of the stem to be implanted would likely be influenced also by the extent of the bone defect, in the current study a single stem diameter was used for all defects in order to facilitate comparisons across the different defect conditions. As the selection of the implant size was driven by the worst case scenario, the current model is likely to overestimate the amount of unloading of the remaining bone stock (stress shielding) for the less critical defect conditions. Further analyses should thus aim to better quantify the influence of stem size on the stress shielding in the remaining bone stock. For such analyses that investigate the mechanical environment of the bone in more detail, however, a more detailed geometrical model of the defect situation would be required.

Although it has been debated that bone support of the proximal part of a revision implant is not necessary [29], concerns about the stresses generated in the implant still exist. To overcome the influences of extended bone defects on implant stresses in the revision stem, distal fixation [13], fluted stems [30], material properties [31], appropriate reconstruction of offset and anteversion have been recommended. The study results supports evidence on the influence of proximal bone support on implant stresses, particularly on the tension side of the implant [32]. The results suggest that key to restorable joint function and to avoid critical implant stresses is to provide distal fixation of the implant during extended bone defect conditions. The simulation results also support clinical evidence of the increased implant survival observed during distal fixation of the implant during revision THR [13,30]. Assessing the conditions in the implant under extreme loading, during uncoordinated activities such as stumbling, when hip contact forces can reach over 8 times body weight [33], was beyond the scope of this study, however, and may pose more of a challenge for the survival of the implant.

Although, to the best of our knowledge, there is no literature on the cortex thickness for the range of defect situations examined in this study, we have modelled a 2 mm thin proximal cortex (based on radiographic observations), by using an equivalent elastic modulus of 5GPa, as confirmed using second moment of area calculations. As a result, the implant stresses calculated using physiological-like loading conditions on the revision prosthesis show no critical stresses that are likely to lead to implant failure. This supports the low rates of fracture reported in clinical studies for the standard Wagner SL stem used in these challenging revision situations [5,9].

The use of an implant with an increased offset is thought to improve the stability of the joint by removing any laxity of the surrounding soft tissues. Changes in the geometry of the reconstructed joint, however, are known to influence the joint contact forces and therefore the implant stresses [19,22,34]. By effectively increasing the lever arm of the one-joint abductor muscles at the hip, the larger offset prosthesis reduces the muscle forces required to balance the varus moment at the hip, and consequently the hip joint contact forces [22]; findings that are in agreement with a simplified experimental study [35]. Despite this likely decrease in the muscle and hip joint contact forces, the present work indicates that increasing the offset can lead to an increase in the implant stresses. From a mechanical perspective, it seems that the influence of the decrease in muscle and joint contact forces, is outweighed by the increased lever arm of the hip joint contact force itself, which is created from a combination of the increased implant offset and the distal anchorage, and actually results in larger bending and torsional forces on the implant. While slight modifications in the neck region of the implant had to be introduced to increase the prosthesis offset the stem was entirely identical between the two implant variants, facilitating the comparison of the stresses within the implant shaft between the two designs. The implementation of geometrical modifications to a clinically successful implant therefore raises the question of whether the benefits of tight soft tissues encapsulating the joint, and therefore a possible improvement in joint function and reduction in the dislocation rate, outweigh the increased risks of implant failure when implanted in a mechanically incompetent femur.

The maximum implant stresses in this study were observed when the increased offset (44 mm) version of the stem was implanted with an anteversion of 4°. Similar stress magnitudes were produced by the configuration of an increased offset and increased anteversion. Whilst a direct validation of the predicted stresses against e.g. in vitro measured conditions would be desirable, current in vitro designs do not allow to represent the complex musculoskeletal loading conditions as used in the current study. In order to ensure that the comparisons of the predicted implant stresses were valid, a convergence analysis in which the element sizing was increased over a number refinements and also the order of the shape function of the elements was varied from linear to non-linear functions, it was ensured that the element sizes were adequate to represent the stress fields within the implanted femurs. Furthermore, we could show that by applying physiological-like boundary conditions (i.e. muscle and joint contact forces as well as physiologically reasonable displacement constraints [27]), the overall deformation of the bone-implant constructs fell within 1 to 2 mm and therefore within the range of experimentally measured data. Lastly, as largely identical meshes of the shaft region of the implants were used in this comparative study design, any systematic error in the modeling process would likely influence the results for all models in a similar manner and would therefore unlikely influence the comparisons.

Since the geometry of the Standardized Femur was used in this study, the loading conditions could only be estimated. However, the methodology has been previously validated against measured *in vivo *hip contact forces in patients [20] and resulted in a complete and balanced set of muscle and joint contact forces. The use of such a balanced force model, together with physiological boundary conditions [27], is essential for analysing loading conditions in the femur [21].

This study has evaluated the stresses in the Wagner revision stem after variations in design (offset) and surgical implantation (anteversion), and establishes an initial understanding of the possible risks that could accompany a modification to the offset of a distally anchored revision stem and variations in its surgical implantation. By considering the extreme case of a type IIIb bone defect, we conclude that when the Wagner stem is used within its prescribed manufacturer's limit the restoration of femoral offset to restore joint function is unlikely to result in stresses that lead to mechanical failure of the implant during routine activities of daily living. These results will need to be confirmed clinically, especially in cases where uncoordinated activities such as stumbling are prevalent.

## Competing interests

The authors declare that they have no competing interests.

## Authors' contributions

MOH co-conceived and participated in the coordination of the study as well as drafting the manuscript. MM performed all finite element analyses of the implanted femur and aided in drafting the manuscript. WRT aided in study conception, provided the musculoskeletal loading conditions and participated in the manuscript preparation. DYK created the solid models and performed first pilot studies to create the finite element meshes, including collection of pilot data and initial analyses into the straining of the intact bone. AS participated in the transfer and application of the musculoskeletal loading conditions onto the finite element models and performed initial analyses of the implanted femur. GND conceived the study and participated in its coordination. CP co-conceived the study, supervised the clinical determination of implant sizing and the implantation of the prosthesis as well as the definition of the defects. He also aided in drafting and approving the manuscript. All authors read and approved the final manuscript.
